# Evaluation of the National Return of unwanted medicines (RUM) program in Australia: a study protocol

**DOI:** 10.1186/s40545-017-0126-6

**Published:** 2017-12-07

**Authors:** Amanda J. Wheeler, Jean Spinks, Emilie Bettington, Fiona Kelly

**Affiliations:** 1 Menzies Health Institute Queensland, Griffith University, Brisbane, Australia; 2 Faculty of Medical and Health Sciences, University of Auckland, Auckland, New Zealand; 3 Centre for Applied Health Economics, School of Medicine, and Menzies Health Institute Queensland, Griffith University, Brisbane, Australia; 4 School of Pharmacy, Menzies Health Institute Queensland, Griffith University, Gold Coast, Australia; 5 School of Human Services, Griffith University, Queensland, Brisbane, 4131 Australia

**Keywords:** Unwanted medicines, Waste, Australia, Medicines disposal, Mixed methods design

## Abstract

**Background:**

The National Return of Unwanted Medicines (NatRUM) program in Australia is one of the few nationally coordinated, free-to-consumer schemes to dispose of unwanted medicines globally. This scheme has been in operation since 1996, however, little is known about public awareness of the scheme and its effectiveness in reducing unsafe disposal practices. The study objectives are to undertake a review of (1) the current use of the NatRUM scheme by consumers; and (11) to investigate disposal practices and beliefs of the general population.

**Methods/design:**

A two-stage, mixed-methods study will be undertaken. Stage One will include a nation-wide audit of a representative sample of unwanted medicine bins, collected by community pharmacies, for incineration. The audit will detail the type and amount of unwanted medicines collected and if they are subsidised on the national formulary (Pharmaceutical Benefits Scheme). Stage Two will include: (i) a large, representative, general population survey; and (ii) more detailed interviews with a sub-set of this sample, who take five or more medications. Results will quantitatively describe the awareness of the NatRUM scheme, disposal practices and the volume of unwanted medicines stored in the home. It will qualitatively describe beliefs and perceptions about storage and disposal practices.

**Discussion:**

It is anticipated that this study will provide valuable insights about how Australians dispose of unwanted medicines, their awareness of the NatRUM scheme and how the scheme might be strengthened. Results will inform the Federal Department of Health and NatRUM Ltd. Board at a local level, as well as other countries who are yet to develop or implement coordinated disposal schemes. A number of challenges are expected, including ensuring the consistency of medicines terminology during the bin audit and recruiting a representative sample of Australians for the general population survey. Results of this study will be widely disseminated to support the translation of findings into practice.

## Background

Global medicine use with both prescription and over-the-counter (OTC) medicines is increasing, and estimated to reach 4.5 trillion doses in 2020; an increase of 24% from 2015 [1]. The implications of this increase are that in 2020 more than half of the world’s population will take more than one dose of medicine per day, and this change will be driven largely by increased use in emerging markets such as India, China, Brazil and Indonesia [1]. Echoing this worldwide trend, at a local level in Australia, prescription volumes have increased 15% over the last five years (2010–2015) to 211.4 million in the year ending June 2015 [2]. Minimising medicines waste and instituting appropriate disposal practices for unwanted medicines (that is, expired medicines, and in-date medicines that are no longer required, including used or unused packs), are consequently a global issue.

Adverse consequences of inappropriate disposal of medicines in landfill and via the sewerage system have been reported, including identity theft from personal information on medicine labels disposed in garbage [3], and concentrations of medicines detectable in surface and drinking water [4, 5]. Similarly, adverse consequences from accumulation of unwanted medicines stored in the home present safety hazards such as accidental ingestion and security issues such as theft [6]. All of these issues pose significant environmental, economic and public health concerns.

Routes for medicines to enter the environment include excretion from the human body (metabolites and parent compound) through urination/defecation, washing off directly from the skin whilst bathing (e.g. creams and ointments), or from inappropriate disposal in the toilet, sink and/or household garbage [3, 7, 8]. Sewerage systems are not equipped to remove medicines and their metabolites effectively and hence these may be discharged into waterways and subsequently into drinking water supplies [9]. Medicines disposed in the garbage end up in landfill and may leach out more slowly into water systems [10]. Once in waterways, medicines and their metabolites may affect plant, marine and animal life, and potentially human life [11, 12]. Future research will determine the degree to which concentrations may be harmful to human health.

As the volume of medicines wastage in communities increases, the environmental and public health effects, and risks outlined here, will be compounded with significant financial implications [13]. Limited health service budgets need cost-effective medicine use. Reducing the volume of unused medicines in the home is a complex issue as highlighted by the 2009 evaluation of NHS medicines wastage in the United Kingdom [14]. The evaluators in their recommendations emphasised the need to focus on improving health outcomes rather than reducing waste alone; opportunities included reducing excessive and irrational prescribing with regular reviews of medication regimens by doctors and pharmacists (e.g. Medicines Use Review programs), targeting adherence support for those starting new medicines, or finishing courses of medicines and reviewing repeat prescribing and dispensing processes [14, 15]. Any changes need to be considered alongside consumer convenience and administrative burden.

Research studies on household disposal practices for unwanted medicines around the world have identified that the most popular methods were pouring them down the toilet or sink, or throwing them in the garbage [3, 16]. A systematic review of global disposal practices found that the method preferred was influenced by the formulation and/or type of medicine: sinks or toilets were favoured for liquids, whilst solid and semi-solid forms such as tablets/capsules and ointments/creams, were disposed of in household garbage. Medicines considered to be more harmful, such as antibiotics, were more often returned to a pharmacy, whereas OTC ‘everyday’ products such as cough medicines were poured down sinks or toilets [16].

The aforementioned systematic review also identified that ‘confusion exists about the proper way of medication disposal’ (pg. 297) in many countries because standardised guidance and protocols on appropriate disposal of unwanted medicines, including access to a nationwide disposal system, were lacking [16]. In contrast, in Sweden, where a national disposal system using pharmacies was established in 1971, 73% of households surveyed, reported that they would use this method for disposing of their unwanted medicines. Only 3% of those surveyed reported disposing of their unwanted medicines in the garbage and none had poured medicines down the drain [17].

In July 1998 the Australian Government also introduced a program that provides a safe, free, and convenient means for the public to return unwanted and out-of-date medicines to community pharmacies; the National Return and Disposal of Unwanted Medicines (NatRUM) scheme. The medicines collected in specific RUM (Return of Unwanted Medicines) bins are then disposed of by high-temperature incineration (which is the Environment Protection Authority (EPA) approved method of disposal) [18]. However, whilst national medicine collection and disposal schemes that are free for the public, such as the Swedish and Australian programs, are becoming more common (e.g. in the United Kingdom and European Union), these are not standard in all countries.

Incineration of medicine waste collected via the Australian NatRUM scheme has increased considerably over time; in the 2015/2016 financial year 705,079 kg of medicine waste was incinerated, an increase of 7.7% compared with the previous year [18]. Two reviews of the NatRUM scheme were undertaken in 2005 and 2013 [19, 20]. The 2005 study interviewed a sample of 605 consumers returning unwanted medicines to community pharmacies in one state of Australia. Most of these consumers had used the NatRUM scheme previously after being informed about it by a pharmacist. The main reasons for consumers to return medicines were: because it had expired (31%), person taking medicine (usually a relative) was deceased (26%), or the medicine (prescription) had been changed (8.5%) [19]. The 2013 study was a medicine wastage audit of a random sample of 686 RUM bins collected from six regions of Australia. Most of the returned medicines were scheduled (e.g. prescription, pharmacist-only, or pharmacy-only medicines), and almost half were still within their expiry date [20]. Six of the 10 most commonly returned medicines were also the most frequently dispensed under the Pharmaceutical Benefits Scheme (PBS).^1^

Despite this increasingly used nation-wide scheme, information about household waste disposal practices from the Australia Bureau of Statistics in 2012 estimated that more than one million Australian households had discarded unwanted medicines, drugs or ointments with their usual garbage [21]. For households who had disposed of these items in the last 12 months, 55% had disposed of them with the usual household garbage and 14% had poured them down the drain or toilet [21].

The present study extends previous work, providing a timely opportunity to explore the current use of the NatRUM scheme and will offer further insight into practices and awareness from a sample of the general public and healthcare workers about this free national scheme, and their attitudes and behaviours towards the storage and disposal of unwanted medicines.

### Study design overview

This mixed-methods study will involve two stages. Stage One will undertake an Australia-wide audit of a representative sample of RUM bins. The objectives of this stage are:

- To quantitatively describe the amount and types of medicines returned to pharmacies using a statistically valid sample of returned RUM bins from all states and territories of Australia; and
- To identify the proportion of waste that is PBS-subsidised and to extrapolate the value of ‘wastage’ to the PBS.

In Stage Two a two-step general population survey will be conducted. Step One will utilise an online household survey with a general population sample. Step Two will invite a sub-set of survey respondents from Step One who take five or more medicines, to participate in a telephone interview. The objectives of Stage Two are:

- To assess general population awareness of: the NatRUM scheme; practices for the disposal of unwanted medicines; and the risks of accumulating medicines;
- To identify general population practices for household disposal of unwanted medicines in the previous 12 months;
- To quantitatively describe the volume of unwanted or expired medicines stored in the households of a sub-set of the general population with higher medication burden; and
- To qualitatively explore the storage and disposal practices of a sub-set of the general population with higher medication burden.

## Methods

### Stage one

The RUM bin audit methodology was adapted from the earlier audit conducted in 2013 [20]. This will allow a number of audit outcomes to be compared to the 2013 outcomes. Amendments will address limitations of the earlier audit, and changes to the NatRUM scheme as it has grown over the intervening period e.g. increased number of incineration sites from one to three.

#### Sample size and RUM bin selection

The design of the audit must ensure that a statistically relevant sample of RUM bins from all Australian states and territories are included (*n* = 8). Over the last three years (2013–2015), data provided by NatRUM Ltd. showed that an average of 10–12,000 bins per month were collected for incineration. For the purposes of calculating the minimum sample size for a statistically valid sample of RUM bins in this study, 12,000 bins per month was used in the Raosoft® sample size calculator (http://www.raosoft.com/samplesize.html); a minimum of 373 bins would need to be sampled (assuming 5% margin of error and 95% confidence interval).

Table 1 outlines the number of RUM bins calculated to ensure an Australian-wide representative sample of 373 bins. Based on the 2013 audit [20], it was estimated that an additional 10% (37 bins) would require inspection to ensure that no bins contained more than 50% as loose tablets/capsules i.e. without any form of packaging (see exclusion criteria listed in *Bin contents and inspection* section).

**Table 1:** Selection of RUM bins required for audit^a^

State/territory	Average number of pallet collections per month	Average number of bin collections per month	Percentage of bins (%)	Number of bins to be audited
Australian Capital Territory	2	96	0.8	3
New South Wales	79	3792	30.4	113
Northern Territory	2	96	0.8	3
Queensland	48	2304	18.5	69
South Australia	37	1776	14.2	53
Tasmania	4	192	1.5	6
Victoria	68	3264	26.2	97
Western Australia (including Christmas Island)	20	960	7.7	29
TOTAL	260	12,480	100	373

^a^ *Pellet Collections by region (first six months of 2015/2016): National data (excluding Western Australia) provided by NatRUM Limited. Data for Western Australia provided by the Western Australia Incineration Site*

Incineration contractors at the three licensed plants (located in Victoria, Queensland and Western Australia) will be requested to randomly select and set aside one bin in every ten from each sequential pallet arriving from all states or territories in the month prior to the audit, until the total (plus 10%) for that site is achieved.

#### Data collection and training

Data collection tools and protocols developed specifically for the 2013 NatRUM audit were reviewed and refined to ensure that the audit will build on, and extend, the evidence base; e.g. whether medicines have been used (opened or full pack) and calculating the proportion of medicine that is returned (compared with 2013 where all medicine packs were treated as unopened and 100% contents were assumed) [20]. Additional variables were identified from a similar audit in New Zealand (DUMP project [22]) and in consultation with the NatRUM Ltd. Board. Table 2 outlines the variables, to be included in the audit. A purpose-built database has been developed in Microsoft Access®. We used Australian Medicines Terminology (AMT) - a subset of the SNOMED CT-AU clinical terminology, supported by the Australian Digital Health Agency (http://www.digitalhealth.gov.au/get-started-with-digital-health/what-is-digital-health/clinical-terminology remove extra line here and rejoin sentence). This national terminology unambiguously identifies branded and generic medicines commonly used in Australia. It includes a list of most prescription and OTC medicines and complementary products sold in Australian pharmacies, allowing generic and/or trade names to be easily searched and data such as form, strength and original quantity to be easily selected. For items not available in this list, manual entry of the name of the product, form, strength and original pack quantity will be required. The database and data collection process have been piloted with three RUM bins at the Queensland incineration plant and a number of modifications were implemented. The Access® database has been loaded onto laptop computers for data entry in real time at each incineration site.

**Table 2:** Variables to be included in RUM audit and post-audit analyses

Characteristics of RUM bins	Source of container (state or territory)
	Container weight
	Percentage filled
Content of RUM bins	Medicine characteristics
	Brand name
	Generic name
	Manufacturer
	Medicine schedule (i.e. unscheduled, S2, S3, S4 or S8)
	Therapeutic category (PBS code)
	Medicine dose
	Form of medicine
	Whether medicine is PBS subsidised or private
	Whether medicine is expired
	Packaging of medicine included
	Evidence of attempted destruction for S8 medicines
	Presence of inappropriate items (e.g. sharps, rubbish)
	Complementary and alternative medicines
	Medicine in a sample package
	Medicines characteristics once dispensed
	Year dispensed^a^
	Quantity dispensed^a^
	Quantity returned^a^
	Evidence of medicine use^a^
	Proportion of dispensed quantity unused^a, b^
	Medicine unlabelled
	Category of medicine dispensing (e.g. concession or general)^a^
	Dose administration aids (DAA)
	Type of DAA
	Proportion of medicines unused in DAA

*PBS = Pharmaceutical Benefits Scheme; S2 = Pharmacy medicine; S3 = Pharmacist-only-medicine; S4 = Prescription medicine; S8 = Controlled medicine*

^a^ *These variables extend the evidence base of data collected during the 2013 audit*

^b^ *Only medicines packaged as foil strips/blisters will be counted and assumptions used in the New Zealand DUMP project audit* [22] *will be applied to documentation of quantity of returned inhalers, creams, liquids*

Audit training tools, including a data collection protocol and quality assurance procedure, and Occupational Health and Safety (OH&S) Standard Operating Procedure (SOP) were developed in consultation with incineration plant managers, personnel from Griffith University OH&S and NatRUM Ltd. (all training tools and protocols are available from correspondence author on request).

Data collection will be conducted at each of the three incineration sites by research assistants, working in pairs (one person inspects and counts whilst the second enters the information into the database), under the supervision of the project Data Collector Manager, (a registered pharmacist). An additional research assistant will be available at each site to act in a quality control role and to provide cover for breaks and/or absences such as unexpected illness. Data collectors will be trained at each site to safely audit a RUM bin and to enter data into the Access® database. Training will include familiarisation with the specific auditing equipment including protective clothing (gloves, safety glasses, dust masks, coveralls), and tools (tongs, tweezers, scales), and a demonstration of how to safely open and assess the contents of a RUM bin. Training will also emphasise the importance of the confidential nature of bin contents including any pharmacy, prescriber and patient information. A representative from Griffith University OH&S will be present for this training.

#### Bin and contents inspection

Each RUM bin will be visually inspected prior to opening to record the source of the bin (state/territory and wholesaler returning the bin), container weight and bin identification number. On opening the bin, the contents are spread over a table and visually inspected for any signs of hazardous materials. If inappropriate items are found, the bin is to be immediately resealed and sent for incineration. RUM bins will be excluded from further assessment if they: are less than 50% full; or contain a high proportion of general waste; or contain more than 50% loose tablets (not in an identifiable bottle/packet or blister/strip); or contain hazardous items such as broken glass, biological waste, unknown liquid or powder waste.

Once it is decided that a bin is suitable for auditing, details of each discarded medicine will be recorded in the database. Information recorded includes the name of the medicine (brand or generic), strength and pack size. The expiry date, schedule, if there is a dispensing label present, dispensing category and year dispensed is then recorded, together with the quantity returned. For items that are a unit dose (i.e. tablets, capsules, nebules, prefilled syringes, etc.), the exact number of units returned is counted. For liquids, the percentage returned is estimated (empty = 0%; 1–25% = 25%; 25–50% = 50%; 50–75% = 75%; and 75–100% = 100%) and creams, gels and ointments are weighed to assess volume remaining. The amount remaining in aerosols is recorded from the dose indicator or estimated by placing the device/canister in water to determine whether it is full (quickly sinks), used (slowly sinks) or empty (floats). Loose tablets and capsules (i.e. those not in bottles or blister packs) and products that are unidentifiable (e.g. from another country or not in original packaging) will be excluded. Volumes of complementary and/or alternative medicines returned will be estimated rather than counted (0–25%, 26–50%, 51–75% or 76–100% full).

If dose administration aids are present, the type (e.g. dosette, blister or sachet pack) is to be recorded and proportion remaining estimated (e.g. 0%–25%, 26%–50%, 51%–75%, and 76%–100% full).

When an item is identified as a Schedule 8 (controlled) medicine, this will be recorded specifically in the database, together with any evidence of destruction (i.e. cut and empty foil strip or an empty bottle).

If any sharps are present, including any used or unused needles and syringes, lancets, and blades [23, 24], this will also be recorded specifically in the database.

A number of quality assurance processes have been built into the audit to ensure safety and adherence to legislation, audit protocols and data entry processes. For example, re-inspection and checking of data entered for the first RUM bin and a second, randomly selected bin for each pair of data collectors.

#### Data analysis

For data collection purposes, *trade product pack terms* are used, allowing data collection personnel to search and select on branded or generic names of products, for example:

- Panadeine Caplet® (codeine phosphate hemihydrate 8 mg + paracetamol 500 mg) tablet: film-coated, 12 tablets.

Use of the AMT allows linkage between trade product pack terms with other formal definitions such as the *medicinal product pack term*:

- paracetamol 500 mg + codeine phosphate hemihydrate 8 mg tablet, 12 tablets; and *the medicinal product term*:
- paracetamol + codeine.

Following data collection the trade product pack terms will be matched with both the medicinal product pack and medicinal product terms (using the SNOMED CT-AU v1.7 Common Release). AMT is also compatible with PBS data and hence the PBS dataset will be merged, along with the Anatomical Therapeutic Chemical (ATC) classification system number, into the bin audit database. This allows PBS items to be matched with a price (Manufacturer’s ‘Ex-Manufacturer Price’ for a manufacturer’s pack).

Automated matching will not be possible for manually entered items (where no trade product pack listing was available at data entry), requiring manual matching in some instances. Following data matching, audit data will be cleaned in preparation for visualisation and descriptive data analyses. Using a number of assumptions an extrapolated value of ‘wastage’ to the PBS will be estimated. This estimate is sensitive to the definition of waste, such as whether only unopened packs are counted, whether products were expired at the time of counting (but not necessarily at the time of disposal), and if a dispensing label was visible. Four assumptions will be tested and presented alongside the main results: i) dispensed, unopened, not expired; ii) dispensed, unopened, expired and not expired; iii) dispensed, opened and unopened, and not expired; and iv) dispensed, opened and unopened, expired and not expired.

### Stage two

#### Step one

The first step of Stage Two is a 10-min online general population survey conducted with a representative sample of the Australian population, aged over 18 years.

##### Participants

An experienced research panel company (Research Now®) have been contracted to undertake the data collection with an existing panel of individuals who represent the Australian adult population. Research Now® will invite participation to achieve a national representative sample based on gender, age and geographical location from the most recent census (2011) data (Table 3) [25].

**Table 3:** General population survey sample characteristics

	Australian representative sample [25] %	Survey sample targets for *N* = 4302 n
Gender		
Female	51	2193
Male	49	2107
Age range (years)		
18–24	12	516
25–34	18	774
35–44	19	817
45–54	18	774
55–64	15	645
65–99	18	774
State or territory		
Australian Capital Territory	2	86
New South Wales	32	1376
Northern Territory	1	43
Queensland	20	860
South Australia	7	301
Tasmania	2	86
Victoria	25	1075
Western Australia	10	430

The most recent Australian Bureau of Statistics survey about waste disposal practices (conducted in March 2012) included data from 12,870 households. This survey used a multi-stage area sampling method [21]. A sample size of 4300 is proposed for our general population survey to enable comparison with the Australian Bureau of Statistics survey results.

##### Survey and data collection

The survey questions were developed using Australian Bureau of Statistics tools (the 2012 Waste Management questions and the 2014–2015 National Health survey [21, 26]), relevant literature [19, 22], and input from the NatRUM Ltd. Board (available on request from correspondence author).

The survey is designed to take around 10 min to complete. The questions have multiple-choice options with a free-text response available when the ‘other’ option is selected. It consists of:

- 11 demographic questions (e.g. age, gender, state/territory, country of birth, language spoken at home, living arrangements, education, employment, household income);
- Four questions on whether respondents currently have unwanted (used, unused or expired) medicines in their home, the type of medicines retained (prescription, OTC, complementary and/or alternative) and why they have retained them;
- Three questions on disposal practices (whether respondents have disposed of any in the last 12 months, how they disposed of them and why). The questions on disposal practices have been included from the Australian Bureau of Statistics with minimal changes to allow for comparison of results [21];
- Two questions on awareness and use of the NatRUM scheme. Respondents who are not aware of the NatRUM scheme are provided with a brief overview of the scheme, and a link to the website (www.returnmed.com.au), and asked if they would use the national scheme in the future:
- Respondents are also asked to indicate whether they are healthcare workers, with a further four questions relating to the advice they give to their patients about medicines disposal.

Finally, two ‘ranking’ questions will assess a potential social marketing strategy designed to inform better use of the NatRUM program. The first question relates to concerns about storing and disposing of unwanted medicines, the second addresses the best media for the promotion of associated public health messages. In the online version of the survey the order of response options for these two questions will be randomised. Survey respondents will be asked to rank the statements in order of most important/effective to least important/effective.

The survey has been piloted (in hardcopy) with two members of the NatRUM Ltd. Board and seven people from the general population with medicines in their homes who were known to the research team. Minor amendments to the wording and question order were subsequently made.

Working with Research Now®, a user-friendly survey which could be completed on-line, or using mobile devices such as smartphones, was developed. For all questions with six or more multiple-choice options the option list is randomised. The online survey has been soft-launched with 48 panel members to test the response algorithms. For the survey proper, Research Now® will invite participation from its panel members to ensure good sample representation and adjust when necessary to ensure targets for under-represented groups (e.g. younger people) are met for the survey sample (*n* = 4300). Survey respondents will be provided with compensation for their time in accordance with terms of their enagagement with Research Now®.

##### Data analysis

All survey responses will be provided by Research Now® in a de-identified Microsoft Office Excel® spreadsheet. Data analysis will be performed using Microsoft Office Excel® 2007 and Stata® v.13. Statistical analyses will be descriptive in nature for the majority of questions. The ranking questions containing the social marketing statements will be analysed using a rank-order logit regression [27], with robust standard errors to mitigate against unknown specification errors. As sociodemographic variables do not vary across the set of possible rankings, and therefore cannot be identified as main effects, interactions with key sociodemographic variables will be tested for joint significance and included in the final model if they reach statistical significance (*p* = 0.05).

#### Step two

In this step a sub-sample of the survey respondents who self-identify as taking five or more medicines (including complementary, alternative and/or OTC medicines) will be invited to participate in a 15-min telephone interview about any unwanted or ‘when required’ medicines they have at home. The structured interviews aim to build on survey findings by exploring the quantity and nature of unwanted or ‘when required’ medicines that may require disposal, and to explore participants’ perceptions related to disposal practices.

##### Participants

We propose to interview around 5% of the survey sample (i.e. 215 participants). Participants will self-select into the interview sample when they indicate they take five or more medicines in a screening question at the conclusion of the main survey. They will be asked to provide their telephone contact details for follow-up by a member of the research team. The research panel company will provide the researchers with a separate Microsoft Office Excel® spreadsheet containing contact information only (i.e. not including any survey response data). Attempts will be made to contact respondents sequentially at a range of times (including daytime, evenings and weekends), however purposive screening may need to be used to achieve a national representative sub-sample based on gender, age and geographical location from the 2011 census data [25].

##### Interview guide and data collection

The development of the telephone interview guide was informed by data collection tools used in the 2005 Victorian study [19], the New Zealand DUMP project [22], and input from members of the NatRUM Ltd. Board (guide available in Appendix Table 4). The guide explores the quantity and nature of unwanted or ‘when required’ (e.g. analgesic) medicines currently stored in the home and key areas related to medicines storage, use and disposal, and perceived associated risks. Participant views on these topics will be explored in more depth via open-ended questions to build on findings from our survey through contextual information. The telephone interview was piloted with six people from the general population who were taking five or more medicines and minor amendments were made.

An interview protocol has been developed to ensure interviewer consistency. The protocol includes text to be read prior to commencing each interview, including background information on the research, statements to obtain verbal confirmation that the participant understands the nature of the research, and verbal consent to record the interview (recordings will be destroyed following transcription and quality checks). Interviews will be conducted by three pharmacist researchers. The senior pharmacist researcher will observe a selection of interviews for both interviewers for the purpose of training and quality assurance. Additional quality assurance processes include debriefs following interviews, transcript and data entry checks.

Interviewers will ask participants to collect and list, or name, all the medicines in their house, and identify which are regularly used, which are unwanted (expired or no longer required), and which are ‘when required’ medicines. Where possible, and when permitted, participants will be invited to provide information for the household, including for other household members. Details of all the unwanted and ‘when required’ medicines including drug name, formulation, amount remaining and expiry date are collected by the interviewer. Participants will also be asked for information about who uses the medicines and how they would normally dispose of them. Additionally, they will be asked how far in advance they fill repeat prescriptions for regularly used medicines and if they would be prepared to pay for the safe disposal of unwanted or expired medicines.

If time permits, and the participant agrees, they will be asked their views on what they think happens to medicines returned to pharmacies, if they have any concerns or issues with returning medicines, and the perceived risks of keeping unwanted medicines at home. Lastly, demographic information will be collected.

Interviews also present opportunities for the pharmacist researchers to provide information about discarding medicines via the participant’s local pharmacy.

##### Data analysis

Data related to medicines in the home will be entered into a purpose-built Microsoft Access® database and descriptive analyses conducted. The interviews will be transcribed verbatim by an external transcribing company known to the research team who will sign a confidentiality agreement as part of their contract. Transcriptions will be quality checked and thematic analysis conducted of the qualitative responses to open-ended questions, by two pharmacist researchers with experience in qualitative research using NVivo 11®. This will involve coding and categorising units of data until themes emerge. Transcripts will be constantly compared and contrasted so that higher-order themes are identified (i.e. constant comparison method [28]). The target number of interview participants is 215, however, data collection will cease once no new information is offered i.e. data saturation has been reached.

### Ethical considerations

The project has been approved by a Human Research Ethics Committee (2016/449/GUHREC). No identifiable data or participant names will be collected in Stage one or in the first step of Stage two. Interview recordings undertaken in Step two will be coded with a unique identifier and transcripts checked to ensure that they do not contain any identifiable information. All audio-recordings will be destroyed once quality checks of the associated transcripts are completed. The data will be stored for the purposes of the research project at the authors’ university on a secure server for a period of five years post-publication of manuscripts using these data, and then destroyed. Only researchers associated with the study will have access to these data, via a secure server. However, the university has academic integrity processes in place and as a result there is the possibility that data integrity could be audited. If this were to happen, the person responsible for this audit may be required to access this dataset.

## Discussion

### Limitations

In Stage one there are both strengths and limitations with using the Australian Medicines Terminology (AMT) as the basis of the bin audit database. Using this terminology ensures consistency in the medicines terms used; however, as PBS data has not yet been fully reconciled with these terms, the matching of PBS data is incomplete. Manual matching will need to be undertaken for the subset of medicines for which matching could not be automated. Further, a proportion of items will have to be manually entered, with a potential for error, and making data cleaning and analysis of this subset more difficult and time consuming. The bin audit data collection process will involve around 15 pairs of data collectors and three additional research assistants: one at each site to work with the Data Collection Manager. This is a practical solution to manage the size and geographical needs of the audit, however it introduces challenges for standardisation. Consequently, we have introduced a number of strategies to minimise this issue which include: standard operating procedures; data collection protocols and training; real-time data entry directly into a database with limited free-text options; oversight and scrutiny by the Data Collection Manager at all sites; and quality checking of medicines audit processes and data entry for all data collectors.

In Stage two the general population survey will be delivered by an experienced panel research company and participation is restricted to people registered with the company and with internet access. Recruitment via a research company may introduce a degree of selection bias. Similarly, survey participants will self-select into the interview process from the survey, on the basis of taking five or more medicines, and not all potential participants will be contactable. Strategies to minimise selection bias include conducting interviews at a range of times including evenings and weekends and screening participants purposively. It may not be possible for participants in multiple occupancy households to provide information for the entire household, potentially resulting in under-reporting of the overall volume of unwanted medicines in that particular home.

### Dissemination and projected impact

This cross-sectional study aims to explore the current use of the National Return of Unwanted Medicines scheme and provide further insight about practices and awareness from the general public and healthcare workers about the scheme, and their attitudes towards the storage and disposal of unwanted medicines. It is anticipated that this study will provide evidence about the level of knowledge held by a representative sample of adult Australians, particularly in relation to appropriate disposal practices for unwanted medicines stored at home. It will provide initial insights into the variable risk perceptions, risk assessment strategies, and medicines use and disposal behaviours amongst consumers, with implications for quality use of medicines and risk of harm.

The study will ultimately reveal opportunities for national and grassroots information campaigns in raising awareness of the NatRUM scheme and the environmental, economic and public health risks associated with inappropriate medicines disposal and accumulation of unwanted medicines at home; targeting healthcare workers and members of the general population, particularly for those who use, or care for people who use, multiple medicines. To ensure the effectiveness of such campaigns it is important that they are informed by input from stakeholders such as consumer and carer organisations, and align with existing quality use of medicine campaigns. In Australia this includes examples such as: the National Prescribing Service’s *Be Medicinewise* week [29]; the Pharmacy Guild of Australia’s *Ask Your Pharmacist* consumer campaign [30]; or the *Choosing Wisely Australia* initiative [31]. These strategies will ideally encourage people to regularly check and clean out their medicines, limit inappropriate medicines use and/or accumulation, and promote appropriate medicines disposal.

In order to support the translation of findings into the anticipated outcomes described here, the research team plan to disseminate findings in late 2017/2018 in scientific journals, at national meetings (e.g. those with a pharmacy, primary care, and drug safety/quality use of medicines focus), and with pharmacy and healthcare media.

## Appendix

**Table 4:** Key interview guide topics

Medicines in the home	Brand and/or generic name and storage location of medicines regularly used, medicines used as required and medicines no longer used.
Unwanted, unused or as required medicines	Formulation, quantity remaining and expiry date; disposal method/s; risks of keeping in the home.
Medicine supply	Frequency of supply, e.g. on-time, in-advance, just-in-case; household member/s who used the medicine; where the medicine was obtained, e.g. pharmacy, supermarket.
Medicine disposal	Self-reported disposal practices, views on and/or concerns related to what happens to medicines returned to a central location, e.g. a pharmacy.
Willingness to pay for safe disposal	Yes/No; explanation.
Demographic information	Gender, age, living arrangements, location, education, employment status, language spoken at home.

## Abbreviations

AMT: Australian Medicines Terminology
ATC: Anatomical Therapeutic Chemical
DAA: Dose Administration Aid
DUMP: Disposal of Unwanted Medicines Properly campaign
EPA: Environment Protection Authority
NatRUM: National Return of Unwanted Medicines scheme
NHS: National Health Service
OH&S: Occupational Health and Safety
OTC: Over-the-counter
PBS: Pharmaceutical Benefits Scheme
RUM: Return of Unwanted Medicines
S2: Pharmacy medicine
S3: Pharmacist-only-medicine
S4: Prescription medicine
S8: Controlled medicine
SOP: Standard Operating Procedure

## Footnotes

1 The Pharmaceutical Benefits Scheme is the mechanism by which the Australian Government subsidises the cost of necessary medicines for Australian consumers. Most of the medicines are dispensed by pharmacists and used by consumers at home.
